# Antimicrobial activity of selected essential oils against *Streptococcus suis* isolated from pigs

**DOI:** 10.1002/mbo3.613

**Published:** 2018-03-24

**Authors:** Fabiana C. de Aguiar, Ana Lucía Solarte, Carmen Tarradas, Inmaculada Luque, Alfonso Maldonado, Ángela Galán‐Relaño, Belén Huerta

**Affiliations:** ^1^ Departamento de Sanidad Animal Universidad de Córdoba International Excellence Agrifood Campus ‘CeiA3’ Córdoba Spain

**Keywords:** antimicrobial activity, essential oils, MIC, *Streptococcus suis*, vapor

## Abstract

The inhibitory potential by contact and vapor of basil, cinnamon, clove, peppermint, oregano, rosemary, common thyme, and red thyme essential oils (EOs) against 20 strains of *Streptococcus suis* was determined by the disk diffusion test. The broth microdilution method was used to determine the minimal inhibitory and minimal bactericidal concentration (MIC and MBC) of the four selected oils. Furthermore, the bactericidal power (ratio MBC/MIC) was calculated. The EOs with the major potential in the disk diffusion method were red thyme, common thyme, oregano, and cinnamon (∅ mean 16.5–34.2 mm), whereas cinnamon did not show vapor activity. In the microdilution test, all the EOs showed notable antimicrobial activity (MIC_90_ and MBC_90_ 312.5–625 μg·ml^−1^) and a strong bactericidal power (ratio = 1). This is the first study that selects essential oils against *S*. *suis*. New studies about the possible synergic effect of EOs with antibiotics and about toxicity and efficacy in in vivo conditions are recommended.

## 1 INTRODUCTION

*Streptococcus suis* is a major gram‐positive swine pathogen associated with a wide variety of pig diseases, such as meningitis, arthritis, bronchopneumonia, endocarditis, polyserositis, and septicemia. In addition, it is a zoonotic agent that causes severe infections in people in close contact with infected pigs or pork‐derived products. According to the capsular polysaccharide, 33 serotypes of *S*.* suis* have been described with serotypes 1–10 (except 6) and 14, 15, 16, ½,and 1/14 being the most prevalent and virulent ones for pigs (Goyette‐Desjardins, Auger, Xu, Segura, & Gottschalk, 2014; Tarradas et al., 2004). The control of the disease is nowadays based on the antibiotic therapy; however, relatively high levels of resistance (up to 85%) to some antimicrobials commonly used in swine, including lincosamides, macrolides, sulfonamides, and tetracycline, has been worldwide documented (Varela et al., 2013). Different strategies to reduce the use of antimicrobials have been proposed and natural products with antimicrobial effects can be an attractive alternative (Manzanilla et al., 2004).

Essential oils (EOs) are extracts of plants prepared by steam distillation and are generally composed of a combination of substances like terpenes, phenolics, aldehydes, or alcohols, the most of which are volatile (Laird & Phillips, 2012). EOs can affect bacterial permeability and survival, either by direct contact or by contact with their vapor (Martinez & Baquero, 2000; Nazzaro, Fratianni, De Martino, Coppola, & De Feo, 2013). The volatile nature of essential oils could also have a direct application in food preservation and surface disinfection (Laird & Phillips, 2012). The complex composition and different mechanisms of action of EOs may be an advantage over other antimicrobials to prevent the development of resistance of pathogenic bacteria (Knezevic et al., 2016; Yap, Yiap, Ping, & Lim, 2014).

Essential oils have an acceptable activity against gram‐positive and gram‐negative bacteria of interest in human and veterinary medicine, such as *Salmonella*,* Escherichia coli*,* Staphylococcus aureus*, and different species of Genus *Streptococcus* of human origin: *S*. *mutans*,* S*. *pyogenes*, and *S*. *salivarius*. In vitro studies have highlighted the activity of oregano, thyme, peppermint, and cinnamon oils against these bacteria (Freires, Denny, Benso, de Alencar, & Rosalen, 2015; Galvão et al., 2012; Sfeir, Lefrancois, Baudoux, Derbre, & Licznar, 2013). Nevertheless, in all those studies, a limited number of clinical isolates have been analyzed, and there are few studies showing susceptibility of *S*.* suis* to EOs.

In this way, the objectives of this study were to evaluate the in vitro activity of eight essential oils, by direct contact and by contact with the oils vapors, against field isolates of *S*.* suis* of the most important serotypes for swine and humans, and to determine the susceptibility of this microorganism to the four oils with the best antimicrobial potential by means of MIC and MBC determination.

## 2 MATERIALS AND METHODS

### 2.1 Bacterial strains

A total of 19 *S*.* suis* strains from Culture Collection of Animal Health Department (University of Cordoba, Spain) and Central Veterinary Institute of Wageningen (Lelystad, Netherlands) were studied. All the strains had been isolated from diseased and healthy carrier pigs submitted to these centers, and belonged to different serotypes (serotypes 1, 2, 3, 4, 8, 9, 24, 25, 28, and 1/14). In addition, the European reference strain of *S*.* suis* (*p* 1/7) was included in this study. *Streptococcus pneumonie* ATCC 49619 reference strain was used as quality control. The selected isolates were stored at −80°C in Microbank^®^ beads (Prolab diagnostics Co., UK) and were grown in Mueller Hinton agar (MHA) supplemented with 5% defibrinated sheep blood (Oxoid Ltd, ES).

### 2.2 Essentials oils

Eight commercial EOs (purity ≥98%) were purchased from Aromium^®^ (Barcelona, ES), the purity and composition of which was determined by the manufacturer. The list of essential oils and their properties are given in Table 1. All the oils were stored at room temperature in the dark prior to testing.

**Table 1:** Essential oils tested and their properties

Essential oil	Common name	Family	Main composition [%]^a^
*Ocimum basilicum*	Basil	Lamiaceae	Estragole [83.34], eucalyptol [3.34], bergamotene [2.58], linalool [0.89]
*Cinnammomum zeylanicum* (bark)	Cinnamon	Lauraceae	Cinnamaldehyde [69.18], linalool [3.19], eugenol [3.03]
*Eugenia caryophyllata*	Clove	Myrtaceae	Eugenol [85–90], eugenyl acetate [5–10], caryophyllene [0–5]
*Mentha piperita*	Peppermint	Lamiaceae	Menthol [50–55], menthone [20–25], eucalyptol [5–10], menthyl acetate [5–10]
*Origanum vulgare*	Oregano	Lamiaceae	Carvacrol [63.01], thymol [10.56], γ‐terpinene [8.11]
*Rosmarinus officinalis*	Rosemary	Lamiaceae	α‐Pinene + α‐thuyene [22.75], 1.8 cineol [20.63], camphor [18.78]
*Thymus vulgaris*	Common thyme	Lamiaceae	Thymol^NA^, p‐cymene^NA^, linalool^NA^
*Thymus zygis*	Red thyme	Lamiaceae	Thymol [46.9], p‐cymene [21.72], γ‐terpinene [9.32], linalool [4.8]

NA, Not available.

a Based on the data provided by manufacturer.

### 2.3 Antimicrobial activity assays

As a preliminary step, the antibacterial activity of the essential oils was determined by the disk diffusion method, following the Clinical and Laboratory Standards Institute guidelines (CLSI VET01‐A4, 2013) for the disk diffusion test with antibiotics, including the modifications proposed for essential oils (Huerta et al., 2016). Briefly, from an overnight culture, a suspension of 1.5 × 10^8^ CFU·ml^−1^ was prepared in sterile saline solution, and inoculated onto a plate of MHA with 5% defibrinated sheep blood. A sterile 6 mm diameter white disk (Oxoid Ltd, ES), previously impregnated with 15 μl of pure essential oil, was placed onto every plate and once sealed with parafilm, plates were incubated in a 5% CO_2_‐enriched atmosphere at 35°C for 20–24 hr.

Furthermore, the effect of the volatile fraction of every EO was studied with the inverted Petri dish method (Maruzzella & Sicurella, 1960; Ross, O'Gara, Hill, Sleightholme, & Maslin, 2001), placing the disk impregnated with 15 μl of each EO in the lid of MHA with 5% defibrinated sheep blood and incubating under the conditions named before.

The antibacterial activity was evaluated by measuring in millimeters the diameter of the inhibitory zone. All experiments were conducted in triplicate and the mean ± *SD* was calculated for each strain. The standard reference penicillin (Sigma Aldrich Co.; Madrid, ES) was used as reference control for the tested bacteria. Inoculum concentration was checked by viable counts in MHA with 5% defibrinated sheep blood.

### 2.4 Determination of minimal inhibitory and bactericidal concentrations

The essential oils with the major antimicrobial activity in the paper disk diffusion assay were selected to determine the minimal inhibitory concentration (MIC) and the minimal bactericidal concentration (MBC), using the broth dilution method for bacteria isolated from animals (CLSI VET01‐A4, 2013). To facilitate the dilution of the oils and the reading of the results, Brain‐Heart Infusion broth supplemented with 0.15% agar was used (Oxoid Ltd, ES) (Mann & Markham, 1998). The microdilution broth technique was performed as follows: double serial dilutions of selected EOs were prepared ranging from 5,000 to 39.0625 μg·ml^−1^; in a 96 well microtiter plate, 100 μl of each EO dilution was mixed with 100 μl of bacterial suspension (10^6^ CFU·ml^−1^). Then, the plate was incubated at 35°C for 20–24 hr under aerobic conditions. Every assay was carried out in triplicate. Positive (oil‐free broth with bacterial inoculum) and negative (oil‐free broth without bacterial inoculum) controls were included (Bajpai, Baek, & Kang, 2012; Huerta et al., 2016). Penicillin G (Sigma Aldrich Co.; Madrid, ES) was used as quality control.

The MIC was the lowest concentration that prevented the visible growth of *S*.* suis*. The MBC was determined by subculturing 10 μl from the last four wells without visible bacterial growth onto MHA with 5% defibrinated sheep blood. After incubation at 35°C for 20–24 hr 5% CO_2_‐enriched atmosphere, MBC was defined as the lowest concentration resulting in a negative subculture or giving presence of only one colony after incubation. All assays were performed in triplicate.

### 2.5 Statistical analysis

A statistical package SPSS 15.0 for Windows (SPSS Inc, USA) was used for the data processing. The diameter of inhibitory zones and MICs and MBCs results were grouped according to oil type and checked for normality by Shapiro–Wilk test.

The repeated‐measures ANOVA test was used for the selection of the four essential oils with the highest antimicrobial potential in the disk diffusion assay, whereas differences between EOs were estimated by comparison of main effects. The results obtained allowed to establish groups of homogeneity depending on the similarity of the antimicrobial activity. MIC and MBC values were treated as ordinal numerical variables. Comparison of the four selected EOs was performed by the nonparametric tests of Friedman and Wilcoxon and allowed the establishment of groups. Significance was set at *p *< .05.

The concentration that inhibited and killed the 50% and 90% of the tested strains (MIC_50_ and MIC_90_, MBC_50_, and MBC_90_, respectively) was determined for the selected EOs. Microcidal power was calculated by MBC/MIC ratio of the previous parameters and interpreted based on the criteria of Schwarz et al. (2010) and Radhakrishnan, Gnanamani, and Mandal (2011).

## 3 RESULTS AND DISCUSSION

### 3.1 Antimicrobial activity of the eight essential oils analyzed

Essential oils and plants extracts have been traditionally used in human medicine for their anti‐inflammatory, antimicrobial, and immunomodulatory properties (Inouye, Takizawa, & Yamaguchi, 2001; Shaaban, El‐Ghorab, & Shibamoto, 2012). Currently, EOs have been authorized as food additives and their antimicrobial activity is analyzed to be used as disinfectants and a possible alternative to the antibiotic therapy in human and veterinary medicine, especially for diseases caused by multidrug‐resistant microorganisms (Laird & Phillips, 2012; Lv, Liang, Yuan, & Li, 2011).

In our study, almost every EO exhibited good antimicrobial activity against *S*.* suis* (Tables 2 and S1). The oils with a significantly higher inhibitory activity were red thyme and common thyme (Ø mean of inhibition zone 34.2 and 33.2 mm, respectively), followed by oregano (Ø mean of inhibition zone 29.4 mm). Cinnamon, peppermint, and clove (Ø mean 16.5, 16.4, and 15.8‐mm, respectively) showed a similar antimicrobial potential, although it was significantly lower than the previous ones. A weak or nonexistent activity of basil and rosemary was observed. It has been possible to establish five homogeneous groups of EOs, according to the results obtained in the disk diffusion test (Tables 2 and S1).

**Table 2:** Average ± standard deviation and range inhibition zone (mm) of disk diffusion and vapor contact tests of the eight tested EOs against 20 isolates of *S*.* suis,* by homogeneity groups

Essential oil		Disk diffusion		Vapor contact	
		Mean ± *SD*	Range	Mean ± *SD*	Range
Groups^a^ I	Red thyme	34.2 ± 8.2	24.3–49.3	25.7 ± 5.1	20.0–42.5
	Common thyme	33.2 ± 7.3	24.0–49.0	25.6 ± 4.4	19.5–37.5
Group II	Oregano	29.4 ± 5.8	22.0–40.3	23.3 ± 3.5	19.0–30.5
Group III	Cinnamon	16.5 ± 5.2	6.0–27.0	0.8 ± 2.6	0.0–10.0
	Peppermint	16.4 ± 6.8	9.0–36.3	7.6 ± 7.9	0.0–22.5
	Clove	15.8 ± 5.0	6.0–28.0	0.8 ± 2.6	0.0–10.0
Group IV	Rosemary	10.3 ± 2.4	6.3–15.7	0.4 ± 1.8	0.0–8.0
Group V	Basil	7.1 ± 1.0	6.0–9.0	0.0 ± 0.0	0.0–0.0

a The homogeneity groups *p* < .05 in the disk diffusion assay.

Red thyme, common thyme, oregano, and cinnamon have showed the best antimicrobial potential in the qualitative assay against the field isolates of *S*.* suis*, including the European reference strain P1/7. According to the classification proposed by Lv et al. (2011), red thyme, common thyme, and oregano would have a strong activity (inhibition zone ≥20 mm) and cinnamon, a moderate one (inhibition zone between 12 and 20 mm). These EOs have also shown a good antimicrobial activity against a wide variety of microbial pathogens involved in different clinical processes of both humans and animals, including other *Streptococcus* species (Fabio, Cermelli, Fabio, Nicoletti, & Quaglio, 2007; Galvão et al., 2012; Sfeir et al., 2013). Taking into account these preliminary results, red thyme, common thyme, oregano, and cinnamon essential oils were selected for the quantitative study.

We also assessed the possible activity of volatile components of these EOs, since they may have a great antimicrobial potential to be used in respiratory pathologies treatment and disinfection of facilities, preventing the formation of biofilms (Inouye et al., 2001; Laird & Phillips, 2012). Good activity was obtained from the volatile fraction of thyme (red and common) and oregano (Ø mean 23.3–25.7 mm), whereas a limited or absent inhibition was observed for peppermint, basil, rosemary, cinnamon, and clove (Tables 2 and S2).

Despite the great vapor activity of thyme (red and common) and oregano, their inhibition zones were slightly lower than those observed in the direct contact assay. The remaining EOs presented weak or nonexistent antibacterial activity, which disagrees with Inouye et al. (2001), who found good activity of cinnamon and moderate activity of rosemary against *Streptococcus pyogenes* and *Streptococcus pneumoniae*. These differences could be related to the volatility and the absorption of the volatile compounds through the agar (Inouye et al., 2001; Maruzzella & Sicurella, 1960).

### 3.2 Minimal inhibitory concentration and minimal bactericidal concentration

Different studies highlight the usefulness of disk diffusion method as a screening test (Lopez, Sanchez, Batlle, & Nerin, 2005). It may present a weak correlation with the quantitative microdilution technique because of heterogeneity of some oils when diffusing through the agar or their different volatility, depending on the chemical composition or external temperature (Hernandez et al., 2005). The dilution method could be more reliable than the disk method with regards to reproducibility and clinical relevance (Inouye et al., 2001).

The susceptibility of all the *S*.* suis* isolates analyzed in this study to each EO was similar, suggesting a very homogeneous behavior of *S*.* suis* against the analyzed essential oils. MIC values ranged from 156.25 to 312.5 μg·ml^−1^ for oregano and common thyme, from 156.25 to 625 μg·ml^−1^ for red thyme, and from 312.5 to 1250 μg·ml^−1^ for cinnamon. The statistical comparison of the distribution of the MIC and the MBC showed significant difference between the oregano and the two thymes (group I) and the cinnamon (group II) (Table S3). These results are in accordance with the classification proposed by Freires et al. (2015): the essential oils with a MIC range of 101‐500 μg·ml^−1^ would have a strong activity and the essential oils with a MIC range 501–1000 μg·ml^−1^ would have a moderate activity.

However, the values determined for MIC_90_ and MBC_90_ only differed in one dilution (312.5 μg·ml^−1^ for oregano, common thyme, and red thyme and 625 μg·ml^−1^ for cinnamon) (Table 3), and the microcidal power was equal to 1.0 for all the EOs, indicating a notable bactericidal activity for the four essential oils tested against *S*.* suis*. Other gram‐positive bacteria (*Streptococcus* spp., *Staphylococcus* spp., *Listeria* spp.), including multiresistant isolates, have also showed a notable antimicrobial activity of oregano and thyme (Fabio et al., 2007; Lv et al., 2011).

**Table 3:** Minimal inhibitory concentration (MIC) 50 and 90 and minimal bactericidal concentration (MBC) 50 and 90 of the selected essential oils against 20 isolates of *S*.* suis*

Essential oil	MIC^a^ (μg·ml^−1^)		MBC^b^ (μg·ml^−1^)	
	MIC_50_	MIC_90_	MBC_50_	MBC_90_
Red thyme	312.5	312.5	312.5	312.5
Common thyme	312.5	312.5	312.5	312.5
Oregano	312.5	312.5	312.5	312.5
Cinnamon	625	625	625	625

a MIC_50_ and MIC_90_: concentration (μg·ml^−1^) that inhibited the growth of 50% (10/20) and 90% (18/20) of the strains.

b MBC_50_ and MBC_90:_ concentration (μg·ml^−1^) that destroyed 50% (10/20) and 90% (18/20) of the strains.

The absence of bacterial resistance described against EOs is considered to be the main advantage of these products in comparison with other antimicrobial agents (Knezevic et al., 2016). Nevertheless, EOs studies in cell cultures show a dose‐dependent cytotoxic effect, described as an increased apoptosis and cellular necrosis (Dusan, Marian, Katarina, & Dobroslava, 2006). The nontoxic concentration described by Fabio et al. (2007) for thyme is near to the minimal inhibitory concentrations (MIC_90_ and MBC_90_) obtained in our study. However, the nontoxic concentration of cinnamon that they publish was lower than our values. Several studies have shown synergistic effect between the essential oils tested in this work and some traditional antimicrobials, with a notable decrease in the effective concentration (Solarte et al., 2017).

In vivo studies in pigs with oregano, thyme, and cinnamon generally describe a significant increase in growth performance without altering the quality of the carcass (Namkung et al., 2004; Simitzis, Symeon, Charismiadou, Bizelis, & Deligeorgis, 2010), which is associated with a beneficial effect on the intestinal microbiota, nutrient absorption, and on the action of digestive enzymes (Zeng, Zhang, Wang, & Piao, 2015). However, in some studies, the potential of these EOs was lower than that of the antimicrobial agents (Neill et al., 2006). A notable difference has been shown in the productive effect of EOs according to the type and origin of the essential oil, the quantity added to feed and some intrinsic and extrinsic factors, including age group, gastric pH, the nutritional status, the diet, or the environmental conditions of the trial (Lan, Li, & Kim, 2016).

This is the first study that selects EOs with antimicrobial activity against several *S*.* suis* strains. The essential oils of oregano, red thyme, common thyme and cinnamon showed a notable in vitro bactericidal activity, by vapor and/or direct contact. The essential oils could be used alone or in combination with antimicrobial agents to control multidrug‐resistant bacteria, although more in vivo studies on the safety and the effect of essential oils are needed.

## CONFLICT OF INTEREST

No conflict of interest declared.
