# Supplementary material for: Antimicrobial activity of selected essential oils against Streptococcus suis isolated from pigs
Source: Microbiologyopen. 2018 Mar 24;7(6):e00613. doi: 10.1002/mbo3.613 (PMC6291787; doi:10.1002/mbo3.613)
Supplement: Supplementary file 1 [file MBO3-7-e00613-s001.doc]

**Table S1. Mean inhibition zone (± standard deviation in mm) of the disc diffusion test of selected EOs against 20 isolates of *S. suis*.**

|  | **Group I** | | **Group II** |  | **Group III** |  | **Group IV** | **Group V** |
| --- | --- | --- | --- | --- | --- | --- | --- | --- |
| Strain | Red thyme | Common thyme | Oregano | Cinnamon | Peppermint | Clove | Rosemary | Basil |
| P1/7 | 39.0 ±1.0 | 38.0 ± 2.7 | 30.7 ± 3.8 | 20.0 ± 1.0 | 20.3 ± 1.2 | 18.3 ± 0.6 | 10.7 ± 0.6 | 7.0 ± 0.0 |
| 638/03 | 25.7 ± 0.6 | 24.0 ± 2.0 | 22.0 ± 1.0 | 14.3 ± 0.6 | 11.7 ± 0.6 | 14.0 ± 0.0 | 9.7 ± 0.6 | 8.0 ± 0.0 |
| 235/02 | 35.7 ± 2.1 | 33.7 ± 2.1 | 33.0 ± 2.0 | 20.0 ± 1.0 | 16.0 ± 1.0 | 18.7 ± 0.6 | 11.7 ± 0.6 | 8.0 ± 0.0 |
| 682/06 | 40.7 ± 3.8 | 37.3 ± 2.3 | 33.7 ± 3.2 | 20.7 ± 1.2 | 14.3 ± 0.6 | 20.3 ± 0.6 | 10.7 ± 0.6 | 7.0 ± 0.0 |
| 123/11 | 29.7 ± 0.6 | 30.7 ± 1.2 | 28.7 ± 0.6 | 18.7 ± 0.6 | 9.0 ± 1.0 | 17.7 ± 0.6 | 6.3 ± 0.6 | 6.0 ± 0.0 |
| 225/00 | 26.3 ± 0.6 | 25.3 ± 0.6 | 29.0 ± 1.0 | 14.0 ± 1.0 | 13.0 ± 1.0 | 14.0 ± 1.0 | 10.0 ± 0.0 | 8.0 ± 0.0 |
| 14/03 | 27.3 ± 0.6 | 29.7 ± 0.6 | 23.3 ± 0.6 | 14.7 ± 1.2 | 12.7 ± 0.6 | 15.0 ± 1.0 | 8.7 ± 0.6 | 8.0 ± 0.0 |
| 636/03 | 31.3 ± 0.6 | 30.7 ± 0.6 | 26.3 ± 0.6 | 16.3 ± 0.6 | 15.3 ± 0.6 | 15.3 ± 0.6 | 8.7 ± 0.6 | 9.0 ± 0.0 |
| 196/05 | 43.3 ± 3.8 | 42.0 ± 3.6 | 40.3 ± 2.5 | 20.0 ± 1.7 | 26.3 ± 5.5 | 20.0 ± 1.0 | 14.3 ± 0.6 | 8.7 ± 0.6 |
| 40/03 | 29.3 ± 0.6 | 28.0 ± 1.0 | 27.0 ± 1.0 | 6.0 ± 0.0 | 22.7 ± 0.6 | 6.0 ± 0.0 | 11.7 ± 0.6 | 6.0 ± 0.0 |
| 160/03 | 32.3 ± 2.3 | 30.7 ± 0.6 | 25.7 ± 0.6 | 19.7 ± 1.5 | 12.0 ± 0.0 | 13.0 ± 2.0 | 10.0 ± 0.0 | 6.3 ± 0.6 |
| 553/05 | 30.3 ± 0.6 | 28.3 ± 0.6 | 25.3 ± 0.9 | 12.7 ± 0.6 | 16.7 ± 0.6 | 13.3 ± 0.6 | 7.7 ± 0.6 | 6.0 ± 0.0 |
| 10/06 | 24.3 ± 1.5 | 26.0 ± 0.0 | 22.7 ± 0.6 | 13.7 ± 0.6 | 12.3 ± 0.6 | 14.7 ± 0.6 | 10.7 ± 0.6 | 7.0 ± 0.0 |
| 233/01 | 45.3 ± 0.6 | 43.7 ± 0.6 | 36.3 ± 0.6 | 22.0 ± 0.0 | 24.0 ± 1.7 | 21.0 ± 1.7 | 12.3 ± 0.6 | 6.7 ± 0.6 |
| 8010 | 49.3 ± 5.9 | 43.3 ± 1.5 | 40.3 ± 3.2 | 27.0 ± 3.6 | 12.7 ± 0.6 | 28.0 ± 2.0 | 12.3 ± 0.6 | 8.0 ± 0.0 |
| 6217 | 34.3 ± 0.6 | 35.7 ± 0.6 | 29.7 ± 0.6 | 13.7 ± 0.6 | 11.0 ± 0.0 | 13.0 ± 0.0 | 8.0 ± 0.0 | 6.0 ± 0.0 |
| 6218 | 49.3 ± 1.2 | 49.0 ± 3.5 | 36.0 ± 1.0 | 18.0 ± 1.0 | 36.3 ± 2.1 | 17.0 ± 1.0 | 15.7 ± 0.6 | 6.0 ± 0.0 |
| 6221 | 39.0 ± 1.0 | 38.0 ± 2.7 | 30.7 ± 3.8 | 20.0 ± 1.0 | 20.3 ± 1.2 | 18.3 ± 0.6 | 10.7 ± 0.6 | 7.0 ± 0.0 |
| 658/02 | 26.3 ± 0.6 | 25.0 ± 1.0 | 25.0 ± 1.0 | 6.0 ± 0.0 | 9.0 ± 0.0 | 6.0 ± 0.0 | 7.3 ± 0.6 | 6.7 ± 0.6 |
| 699/02 | 25.0 ± 0.0 | 25.7 ± 0.6 | 22.7 ± 0.6 | 12.0 ± 0.0 | 12.3 ± 0.6 | 12.7 ± 0.6 | 8.00 ± 0.0 | 6.0 ± 0.0 |
| $\overline{x}\pm SD$ | 34.2 ± 8.2 | 33.2 ± 7.3 | 29.4 ± 5.8 | 16.5 ± 5.2 | 16.4 ± 6.8 | 15.8 ± 5.0 | 10.3 ± 2.4 | 7.1 ± 1.0 |
| Range | 24.3 – 49.3 | 24.0 – 49.0 | 22.0 – 40.3 | 6.0 – 27.0 | 9.0 – 36.3 | 6.0 – 28.0 | 6.3 – 15.7 | 6.0 – 9.0 |

* The homogeneity groups *P* <0.05

**Table S2.** Mean inhibition zone (± standard deviation in mm) of the vapour contact test of selected EOs against 20 isolates of *S. suis*.

| Strains | Red thyme | Common thyme | Oregano | Cinnamon | Peppermint | Clove | Rosemary | Basil |
| --- | --- | --- | --- | --- | --- | --- | --- | --- |
| P1/7 | 21.5 ± 0.7 | 24.5 ± 0.7 | 22.0 ± 0.0 | 0.0 ± 0.0 | 0.0 ± 0.0 | 0.0 ± 0.0 | 0.0 ± 0.0 | 0.0 ± 0.0 |
| 638/03 | 24.5 ± 0.7 | 23.0 ± 0.0 | 21.0 ± 0.0 | 0.0 ± 0.0 | 9.5 ± 0.7 | 0.0 ± 0.0 | 0.0 ± 0.0 | 0.0 ± 0.0 |
| 235/02 | 22.0 ± 0.0 | 20.5 ± 0.7 | 19.0 ± 1.4 | 0.0 ± 0.0 | 19.0 ± 1.4 | 0.0 ± 0.0 | 0.0 ± 0.0 | 0.0 ± 0.0 |
| 682/06 | 28.5 ± 0.7 | 24.5 ± 0.7 | 22.5 ± 0.7 | 0.0 ± 0.0 | 6.0 ± 0.0 | 0.0 ± 0.0 | 0.0 ± 0.0 | 0.0 ± 0.0 |
| 123/11 | 23.0 ± 0.0 | 24.0 ± 1.4 | 20.5 ± 0.7 | 0.0 ± 0.0 | 0.0 ± 0.0 | 0.0 ± 0.0 | 0.0 ± 0.0 | 0.0 ± 0.0 |
| 225/00 | 23.5 ± 0.7 | 25.0 ± 0.0 | 20.0 ± 0.0 | 0.0 ± 0.0 | 0.0 ± 0.0 | 0.0 ± 0.0 | 0.0 ± 0.0 | 0.0 ± 0.0 |
| 14/03 | 24.5 ± 0.7 | 25.0 ± 0.0 | 20.0 ± 0.0 | 0.0 ± 0.0 | 0.0 ± 0.0 | 0.0 ± 0.0 | 0.0 ± 0.0 | 0.0 ± 0.0 |
| 636/03 | 29.0 ± 1.4 | 28.5 ± 0.7 | 23.5 ± 0.7 | 0.0 ± 0.0 | 6.0 ± 0.0 | 0.0 ± 0.0 | 0.0 ± 0.0 | 0.0 ± 0.0 |
| 196/05 | 26.5 ± 0.7 | 25.0 ± 0.0 | 26.0 ± 0.0 | 6.0 ± 0.0 | 0.0 ± 0.0 | 0.0 ± 0.0 | 0.0 ± 0.0 | 0.0 ± 0.0 |
| 40/03 | 24.0 ± 0.0 | 23.5 ± 0.7 | 23.0 ± 0.0 | 0.0 ± 0.0 | 20.5 ± 2.1 | 0.0 ± 0.0 | 0.0 ± 0.0 | 0.0 ± 0.0 |
| 160/03 | 24.5 ± 2.1 | 26.0 ± 1.4 | 24.5 ± 0.7 | 0.0 ± 0.0 | 16.5 ± 0.7 | 0.0 ± 0.0 | 0.0 ± 0.0 | 0.0 ± 0.0 |
| 553/05 | 27.5 ± 0.7 | 27.5 ± 0.7 | 24.5 ± 0.7 | 0.0 ± 0.0 | 15.0 ± 0.0 | 0.0 ± 0.0 | 0.0 ± 0.0 | 0.0 ± 0.0 |
| 10/06 | 20.0 ± 1.4 | 21.0 ± 0.0 | 19.0 ± 0.0 | 0.0 ± 0.0 | 7.0 ± 1.4 | 0.0 ± 0.0 | 0.0 ± 0.0 | 0.0 ± 0.0 |
| 233/01 | 28.0 ± 1.4 | 27.5 ± 0.7 | 25.5 ± 0.7 | 0.0 ± 0.0 | 9.5 ± 0.7 | 0.0 ± 0.0 | 0.0 ± 0.0 | 0.0 ± 0.0 |
| 8010 | 27.5 ± 0.7 | 26.0 ± 0.0 | 29.0 ± 1.4 | 0.0 ± 0.0 | 15.0 ± 7.1 | 0.0 ± 0.0 | 8.0 ± 0.0 | 0.0 ± 0.0 |
| 6217 | 42.5 ± 0.7 | 35.5 ± 0.7 | 30.5 ± 0.7 | 10.0 ± 0.0 | 6.0 ± 0.0 | 10.0 ± 0.0 | 0.0 ± 0.0 | 0.0 ± 0.0 |
| 6218 | 22.0 ± 0.0 | 26.0 ± 1.4 | 20.0 ± 0.0 | 0.0 ± 0.0 | 0.0 ± 0.0 | 0.0 ± 0.0 | 0.0 ± 0.0 | 0.0 ± 0.0 |
| 6221 | 32.0 ± 0.0 | 37.5 ± 0.7 | 30.5 ± 3.5 | 0.0 ± 0.0 | 22.5 ± 3.5 | 6.0 ± 0.0 | 0.0 ± 0.0 | 0.0 ± 0.0 |
| 658/02 | 20.0 ± 0.0 | 19.5 ± 2.1 | 22.5 ± 0.7 | 0.0 ± 0.0 | 0.0 ± 0.0 | 0.0 ± 0.0 | 0.0 ± 0.0 | 0.0 ± 0.0 |
| 699/02 | 23.0 ± 0.0 | 22.5 ± 0.7 | 22.5 ± 0.7 | 0.0 ± 0.0 | 0.0 ± 0.0 | 0.0 ± 0.0 | 0.0 ± 0.0 | 0.0 ± 0.0 |
| $\overline{x}\pm SD$ | 25.7 ± 5.1 | 25.6 ± 4.4 | 23.3 ± 3.5 | 0.8 ± 2.6 | 7.6 ± 7.9 | 0.8 ± 2.6 | 0.4 ± 1.8 | 0.0 ± 0.0 |
| Range | 20.0 – 42.5 | 19.5 – 37.5 | 19.0 – 30.5 | 0.0 – 10.0 | 0.0 – 22.5 | 0.0 – 10.0 | 0.0 – 8.0 | 0.0 – 0.0 |

**Table S3.** Minimal Inhibitory Concentration (MIC μg ml^-1^) and Minimal Bactericidal Concentration (MBC μg ml^-1^) obtained in the quantitative study of the four best oils, by groups of homogeneity (*P* < 0.05) against 20 isolates of *S. suis*.

|  | **Group I** | | | | | | **Group II** | |
| --- | --- | --- | --- | --- | --- | --- | --- | --- |
|  | Oregano | | Common thyme | | Red thyme | | Cinnamon | |
| Strains | MIC | MBC | MIC | MBC | MIC | MBC | MIC | MBC |
| P1/7 | 156.25 | 312.5 | 156.25 | 156.25 | 312.5 | 312.5 | 625 | 625 |
| 638/03 | 156.25 | 312.5 | 156.25 | 156.25 | 156.25 | 312.5 | 625 | 625 |
| 235/02 | 312.5 | 312.5 | 312.5 | 312.5 | 312.5 | 312.5 | 625 | 625 |
| 682/06 | 156.25 | 156.25 | 156.25 | 312.5 | 312.5 | 312.5 | 625 | 625 |
| 123/11 | 312.5 | 312.5 | 312.5 | 312.5 | 312.5 | 312.5 | 625 | 1250 |
| 225/00 | 312.5 | 312.5 | 312.5 | 312.5 | 312.5 | 312.5 | 625 | 625 |
| 14/03 | 312.5 | 312.5 | 312.5 | 312.5 | 312.5 | 312.5 | 625 | 625 |
| 636/03 | 156.25 | 312.5 | 156.25 | 156.25 | 312.5 | 312.5 | 312.5 | 625 |
| 196/05 | 156.25 | 156.25 | 156.25 | 156.25 | 156.25 | 156.25 | 312.5 | 312.5 |
| 40/03 | 156.25 | 156.25 | 156.25 | 156.25 | 156.25 | 156.25 | 312.5 | 312.5 |
| 160/03 | 156.25 | 156.25 | 312.5 | 312.5 | 312.5 | 312.5 | 312.5 | 312.5 |
| 533/05 | 156.25 | 156.25 | 156.25 | 156.25 | 156.25 | 156.25 | 312.5 | 312.5 |
| 10/06 | 312.5 | 312.5 | 312.5 | 312.5 | 312.5 | 312.5 | 1250 | 1250 |
| 233/01 | 312.5 | 312.5 | 312.5 | 312.5 | 312.5 | 312.5 | 625 | 625 |
| 8010 | 312.5 | 312.5 | 312.5 | 312.5 | 312.5 | 312.5 | 625 | 625 |
| 6217 | 312.5 | 312.5 | 312.5 | 312.5 | 312.5 | 312.5 | 625 | 625 |
| 6218 | 312.5 | 312.5 | 312.5 | 312.5 | 312.5 | 312.5 | 625 | 625 |
| 6221 | 312.5 | 312.5 | 312.5 | 312.5 | 625 | 625 | 625 | 625 |
| 658/02 | 312.5 | 312.5 | 312.5 | 312.5 | 312.5 | 312.5 | 625 | 625 |
| 699/02 | 312.5 | 312.5 | 312.5 | 312.5 | 312.5 | 625 | 625 | 625 |
| Value _50*_ | 312.5 | 312.5 | 312.5 | 312.5 | 312.5 | 312.5 | 625 | 625 |
| Value _90*_ | 312.5 | 312.5 | 312.5 | 312.5 | 312.5 | 312.5 | 625 | 625 |

*MIC_50_ and MBC_50_: concentration (μg ml^-1^) that inhibited and destroyed 50% (10/20) of the strains. MIC_90_ and MBC_90_: concentration (μg ml^-1^) that inhibited and destroyed 90% (18/20) of the strains.
